# Necrotizing Fasciitis Caused by Edwardsiella tarda in an Elderly Patient Without Known Risk Factors: A Case Report and Literature Review

**DOI:** 10.7759/cureus.88295

**Published:** 2025-07-19

**Authors:** Koichi Zokumasu, Hirotaka Tamazawa, Shigeru Abe

**Affiliations:** 1 Emergency Medicine, Kantō Central Hospital, Tokyo, JPN

**Keywords:** bacteremia, edwardsiella tarda, infectious disease pathology, necrotizing soft-tissue infection, septic shock

## Abstract

This report describes the case of bacteremia originating from necrotizing soft tissue infection caused by *Edwardsiella tarda* in an 83-year-old man without typical risk factors, such as hepatobiliary disease or aquatic exposure. The patient presented with extensive necrosis of the right lower extremity that rapidly progressed to septic shock and multiorgan failure despite prompt surgical intervention and intensive care. This case highlights the potential severity of soft tissue infections caused by* E. tarda *and the need for early recognition and aggressive management.

## Introduction

*Edwardsiella tarda*, a facultative anaerobic gram-negative rod-shaped bacterium, causes systemic infections in a wide range of animal hosts, typically in aquatic animals. Although *E. tarda* has historically been noted to cause infections in aquaculture, such as farmed eels, reports of human infections have increased in recent years [1]. Because *E. tarda *is rarely detected in the feces of healthy individuals, it is not considered a normal component of human intestinal flora [2].　

Transmission is generally associated with the consumption of inadequately cooked seafood or direct exposure to fish or seawater. It most commonly causes intestinal infections in humans, although extraintestinal infections have also been reported [3]. Soft tissue infections are less frequently reported and usually occur in individuals with underlying malignancies, chronic liver disease, or immunocompromised states [4]. Necrotizing fasciitis is a rapidly progressive soft tissue infection with high morbidity and mortality. Herein, we present a case of *E. tarda-related* necrotizing fasciitis in an elderly patient, without these commonly recognized risk factors. Given the rarity of extraintestinal presentations, especially those involving soft tissue, further accumulation of clinical experience may enhance the understanding of the full spectrum of *E. tarda* infections.

## Case presentation

An 83-year-old man with impaired mobility was transported from his home to our emergency department. He had been receiving pharmacological treatment for chronic heart failure secondary to atrial fibrillation from his home-care physician. Although the patient had a history of cerebral infarction more than a decade earlier, he was ambulatory with the aid of a walker. He lived with his wife and two sons.

**Figure 1:**
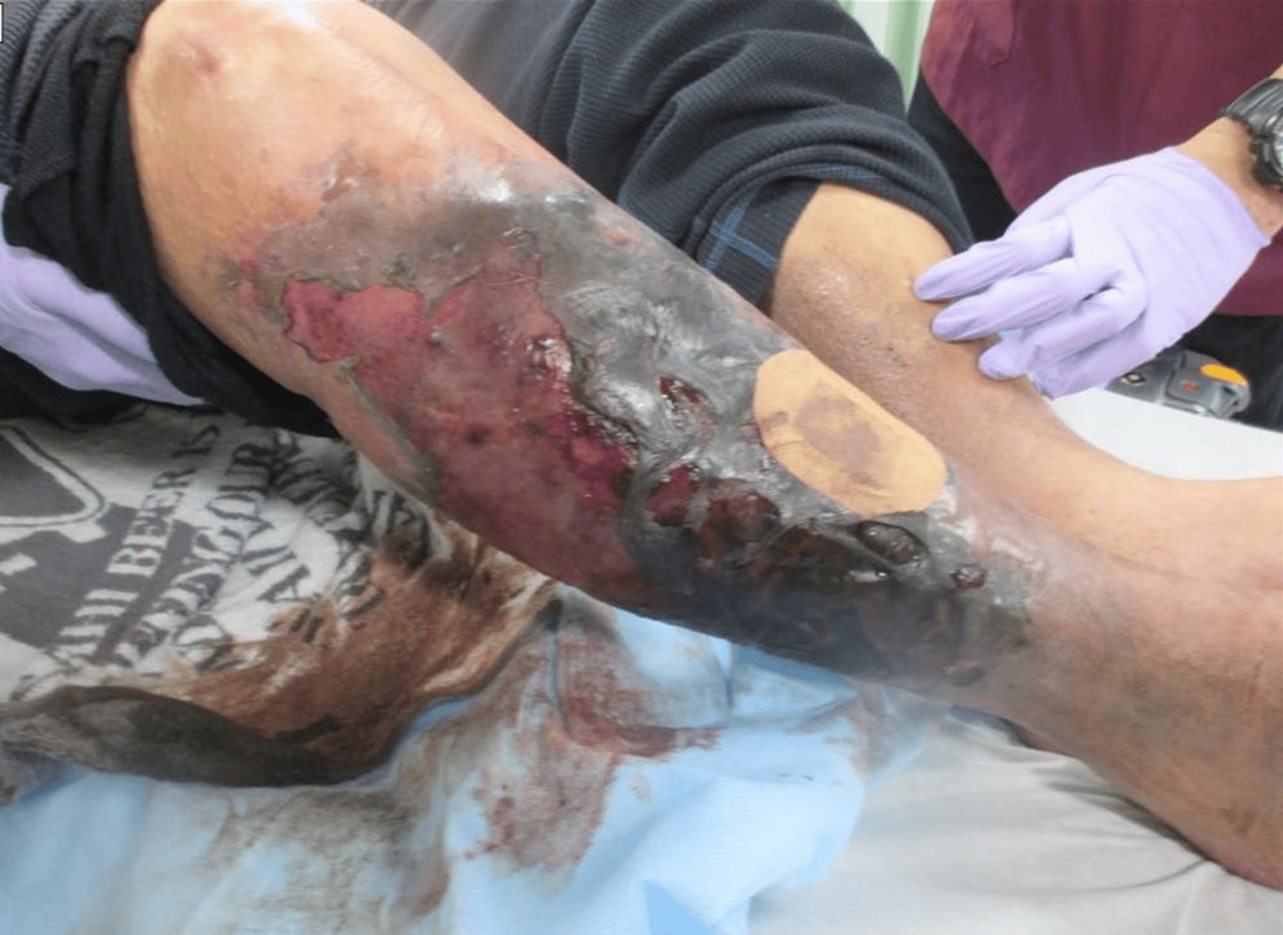
Necrosis and erythema of the right lower extremity

On arrival, the patient was alert and oriented, with no obvious motor paralysis in any of the extremities. He complained of severe pain in his right lower limb and was unable to move because of pain. Upon inspection, erythema was noted from the right inguinal region to the lower extremity, and the lateral aspect of the lower extremity showed areas of black eschar, suggestive of skin necrosis (Figure 1).

According to the patient's family, he had experienced recurrent skin lesions on the right lower extremity for approximately a year; however, no one had recently observed the condition of his leg, and it was unclear when the necrosis had begun. The patient’s vital signs were as follows: body temperature, 36.4 °C; blood pressure, 91/70 mmHg; pulse rate, 132 beats/minute, irregular rhythm; respiratory rate, 16 breaths/minute; peripheral oxygen saturation (SpO₂), 97% on room air. 

Laboratory tests on admission revealed leukocytosis, thrombocytopenia, elevated C-reactive protein, and increased serum lactate. Serum glucose level was within the normal range (Table 1).

**Table 1:** Laboratory data on admission

Parameter	Patient value	Reference range
White Blood Cell ✕10^3^	12.8	6.5 ± 3.0
Stab cell %	2.0	
Segmented cell %	90.0	
Red Blood Cell ✕10^4^/μL	573	540 ± 60
Hemoglobin g/dL	17.1	16.0 ± 2.0
Platelet ✕10^4^/μL	6.1	13.0 – 40.0
Aspartate aminotransferase (AST) IU/L	46	8 – 38
Alanine aminotransferase (ALT) IU/L	20	4 – 44
Blood Urea Nitrogen (BUN) mg/dL	54	8 – 20
Creatinine mg/dl	1.79	0.60 – 1.10
Sodium mEq/L	140	135 - 147
Potassium mEq/L	3.3	3.3 – 4.8
C reactive protein (CRP) mg/dL	39.52	0 – 0.30
Glucose mg/dL	76	70 - 109
Lactate mmol/L	2.7	0.5 - 1.5

A diagnosis of septic shock due to severe soft tissue infection was made, based on hypotension, elevated serum lactate. Two sets of blood cultures and swab samples obtained from three different sites on the wound surface were subjected to microbiological testing. The patient was admitted to the intensive care unit (ICU) with aggressive fluid resuscitation, circulatory support with norepinephrine and vasopressin, and antimicrobial therapy with meropenem and vancomycin.

On day 2 of hospitalization, the patient was transferred to a tertiary care center. Shortly after transfer, all swab cultures and two sets of blood cultures obtained at our hospital returned positive results for *E. tarda*. The clinical course following transfer to a tertiary emergency center was highly complex. On the day of transfer, the patient underwent right knee disarticulation. On day 8, he experienced cardiopulmonary arrest, for which resuscitative measures were initiated, resulting in a return of spontaneous circulation (ROSC). Because residual necrosis developed in the remaining tissue at the stump site, additional debridement and partial resection of the femur were required. The patient subsequently underwent right thigh amputation on day 10. Following amputation, treatment was continued with intra-soft-tissue antibiotic perfusion (iSAP) and negative-pressure wound therapy (NPWT). On day 22, the patient developed airway obstruction due to mucous plugging, necessitating emergency endotracheal intubation. The patient later developed aspiration pneumonia and was dependent on mechanical ventilation. Tracheostomy was performed on day 29 because of the difficulty in weaning. Progressive multiorgan failure ensued, and renal replacement therapy was initiated.

As his overall condition continued to deteriorate, a decision was made in consultation with the intensive care team and his family to pursue palliative treatment at our hospital closer to his home. The patient was transferred to our hospital on day 51. Upon readmission, he demonstrated a marked tendency toward disseminated intravascular coagulation (DIC). On day 54, the patient developed hemorrhagic shock triggered by massive bloody stool. Although the initial resuscitation with fluid and blood transfusions was successful, the patient experienced recurrent bloody stools on day 56, resulting in another episode of shock. A do-not-attempt-resuscitation order was established in consultation with the patient’s family, and the patient died on the same day.

## Discussion

*E. tarda*, a member of the Enterobacteriaceae family, is a facultative anaerobic gram-negative bacterium that primarily infects aquatic animals and rarely causes human diseases. Approximately 80% of human cases present with intestinal infections, such as gastroenteritis, whereas the remaining 20% involve extraintestinal sites, including the hepatobiliary system, central nervous system, and soft tissue [3]. 

Bacteremia due to* E. tarda* is particularly rare. In a surveillance study conducted over 12 years involving 182,668 blood culture sets, only 40 (0.02%) yielded *E. tarda *[5]. Bacteremia is often observed in patients with underlying hepatobiliary disease, malignancy, or immunocompromised states [4]. Conversely, among the reported* E. tarda *infections, it is estimated that less than 5% result in bacteremia or sepsis [6]. Progression from intestinal *E. tarda* infection to bacteremia is uncommon [4]. Previous reports have shown that bacteremia is associated with a high case fatality rate. A 2015 review by Hirai et al. summarized global case reports and reported a case fatality rate exceeding 40% for *E. tarda* bacteremia, with soft tissue infections demonstrating an even higher case fatality rate of 61.1% [6].

However, in recent years, several reports have suggested that the case fatality rate from *E. tarda* bacteremia may be declining. Kamiyama et al. reported a 90-day case fatality rate of only 27% in a retrospective review of bacteremia cases between 2005 and 2016 [5]. In our institution, we identified eight cases of *E. tarda* bacteremia over the past decade (2014-2024), six of which resulted in discharge, and two of them died (case fatality rate, 25%) (Table 2).

**Table 2:** Summary of Edwardsiella tarda bacteremia cases at our institution (2015–2025)

Age (years)	Sex	Diagnosis	Comorbidities	Outcome
85	Female	Cholangitis	Parkinson's Disease	Died
83	Male	Cellulitis	Hypertension	Died
78	Male	Cholangitis	Gastric cancer	Survived
92	Female	Cholangitis	Bronchial asthma	Died
76	Male	Ileocecal inflammation	Unremarkable	Survived
87	Female	Cholangitis	Diabetes mellitus, Ampullary cancer	Survived
91	Male	Unknown fever	Rheumatoid Arthritis	Survived
84	Male	Cholangitis	Cholangiocarcinoma, Prostate cancer	Survived

These observations may reflect improvements in the management of sepsis, including the implementation of early goal-directed therapy and bundled care strategies, rather than improvements in pathogen-specific treatments or changes in the pathogenicity of *E. tarda *[7]. 

Despite the overall decline in case fatality rate from *E. tarda* bacteremia, cases originating from soft tissue infections remain particularly lethal. A literature review of 59 case reports of* E. tarda* infection published between 2015 and 2024 (We searched PubMed using the keyword “Edwardsiella tarda” and reviewed case reports) identified nine cases of bacteremia caused by soft tissue infections. Among the five reported cases of soft tissue infection with bacteremia [8-12], three resulted in death, suggesting a case fatality rate of approximately 60% (3/5) for this subset. In contrast, among the nine cases of bacteremia originating from hepatobiliary infections [13-21], all eight cases with available outcome data survived. Similarly, among the six reported cases of *E. tarda* bacteremia secondary to enteritis [22-27], only one death was documented, corresponding to a 17% (1/6) case fatality rate. 

Table 3 shows the outcomes of *E. tarda* bacteremia distributed according to primary infection location. Notably, the median age of patients was 70 years for those with hepatobiliary infections, 26 years for those with enteritis, and 58 years for those with soft tissue infections. Despite the younger age of the soft tissue infection group, the associated case fatality rate was higher than that in those with hepatobiliary infections.

**Table 3:** Outcomes of Edwardsiella tarda bacteremia stratified by infection site Summary of reported case outcomes based on infection origin. Soft tissue infections showed a higher case fatality rate compared to enteritis and hepatobiliary sources.

Primary Infection Site	Survived	Died	Unknown	Total
Soft Tissue	2	3	0	5
Hepatobiliary	8	0	1	9
Enteritis	5	1	0	6

The specific reasons why *E. The tarda-related* soft tissue infections tend to follow a more severe course remains unclear. *E. tarda* expresses known virulence factors, such as TraT, which confers complement resistance, and type III and VI secretion systems (T3SS/T6SS), which facilitate immune modulation [3,28]. These elements are likely to be involved in both intestinal and extraintestinal infections. They may contribute to overall pathogenicity, but do not explain the difference in severity. 

Interestingly, antimicrobial resistance did not appear to be a major contributing factor to the severity of *E. tarda* infection. Of the 28 reported cases of *E. tarda* infection in the past decade with available susceptibility data [11,14,19,22,29-31], 20 were pan-sensitive. Reported resistant strains have been shown to be resistant to ciprofloxacin, ampicillin, and trimethoprim-sulfamethoxazole. However, none of these cases involved multidrug-resistant strains that would significantly hinder the selection of effective antimicrobial therapy. Furthermore, these resistant cases were not limited to a single clinical presentation, including one case of enteritis, three of soft tissue infection, three of hepatobiliary infection, and one of another. This distribution suggests that antimicrobial resistance is not uniquely associated with soft tissue infections; therefore, antimicrobial resistance alone is unlikely to account for the higher case fatality rate observed in *E. tarda* bacteremia originating from soft tissue infections compared to those of other origins.

As the present discussion is based on a limited number of reported cases, definitive conclusions cannot yet be drawn, and further accumulation of clinical data is essential to fully elucidate the pathophysiology and prognostic implications of *E. tarda* infections. Nevertheless, given the currently available evidence, clinicians should remain vigilant regarding the potential severity of *E. tarda* soft tissue infections, even in patients without classical risk factors, and approach such cases with heightened caution. Finally, although the patient lived with his family and was regularly seen by a home care physician, no one noticed the skin lesion until it had progressed to necrosis. This case serves as an important reminder for clinicians of the need to perform thorough physical examinations and always assess the entire body.

## Conclusions

*E. tarda* is an uncommon human pathogen, and bacteremia originating from necrotizing soft tissue infections remains extremely rare. This case demonstrates that *E. tarda* infections can result in severe sepsis, even in patients without typical risk factors such as hepatobiliary disease or aquatic exposure.

Clinicians should include *E. tarda *in the differential diagnosis of rapidly progressive soft tissue infections and remain vigilant in initiating early surgical consultation and appropriate antimicrobial therapy. Further accumulation of case data is essential to better understand the pathophysiology and prognosis of these infections.
